# Hospital Nurse Perspectives on Barriers and Facilitators to Caring for Socially Disadvantaged Patients

**DOI:** 10.1001/jamanetworkopen.2025.12397

**Published:** 2025-06-06

**Authors:** J. Margo Brooks Carthon, K. Jane Muir, Lee Ang, Kelvin Amenyedor, Daniela Golinelli, Shelli Feder, Ann Kutney-Lee

**Affiliations:** 1 Center for Health Outcomes and Policy Research, School of Nursing, University of Pennsylvania, Philadelphia; 2 The Leonard Davis Institute of Health Economics, University of Pennsylvania, Philadelphia; 3 Department of Emergency Medicine, Perelman School of Medicine, University of Pennsylvania, Philadelphia; 4 Mixed Methods Research Lab, University of Pennsylvania, Philadelphia; 5 Department of Internal Medicine, Yale University School of Medicine, New Haven, Connecticut; 6 Yale University School of Nursing, New Haven, Connecticut; 7 Corporal Michael J. Crescenz Department of Veterans Affairs Medical Center, Philadelphia, Pennsylvania

## Abstract

**Importance:**

Patients from socially disadvantaged backgrounds experience disproportionately worse health care outcomes. Nurses provide most care to patients in hospitals and are informants of health care quality and equity.

**Objective:**

To understand what hospital nurses say helps or hinders their ability to provide quality care to socially disadvantaged populations.

**Design, Setting, and Participants:**

This qualitative study involved a directed content analysis of open-text responses from the RN4CAST-NY/IL survey, which was conducted between April and June 2021. Participants were registered nurses licensed to work in 58 New York and Illinois hospitals identified as high-performing (25 hospitals) and low-performing (33 hospitals) for COVID-19 mortality outcomes in 2021 from a larger quantitative study. The Social Ecological Model informed the study codebook; inductive and deductive coding of the data and content analysis were conducted from March to October 2024.

**Exposure:**

Direct care hospital nurses who participated in the RN4CAST-NY/IL survey.

**Main Outcomes and Measures:**

Open-text responses were from nurses who answered the survey question, “What helps (or hinders) your ability to provide quality care to vulnerable populations? (e.g. low SES, housing insecurity/homeless, racial/ethnic minorities, immigrant, limited English proficiency)?”

**Results:**

A total of 1084 nurses (mean [SD] age, 47.1 [18.2] years) responded to the survey. Most respondents identified as female (986 respondents [91.0%]) and were staff or direct care nurses (765 respondents [70.6%]) with at least a bachelor’s degree (968 respondents [89.6%]). With regard to race and ethnicity, 127 respondents (11.8%) were Asian, 156 (14.5%) were Black or African American, 89 (8.3%) were Hispanic, 693 (64.2%) were White, and 97 (8.9%) were other races. They had a mean (SD) of 18.9 (14.0) years of experience. Six themes described what helped or hindered quality care: (1) profits over patients, (2) care continuity and hospital-community partnerships, (3) insufficient staffing and time constraints, (4) technology to address language barriers, (5) patients’ determinants of health, and (6) individual nurses’ beliefs and backgrounds. Nurses proposed improving health care workforce diversity, strengthening community resources for patients, and advancing tailored cultural competency education as solutions to improve care for socially disadvantaged patients.

**Conclusions and Relevance:**

In this qualitative study and directed content analysis of 1084 open-text responses, nurses identified systemic, institutional, community, and individual clinician-level approaches to improve care for socially disadvantaged populations for equitable care delivery. Nurses’ direct care experiences can inform hospital strategies to improve care for this population.

## Key Points

**Question:**

What do hospital nurses say helps or hinders their ability to provide quality care to socially disadvantaged patients?

**Findings:**

This qualitative, directed content analysis of open-text responses from 1084 hospital nurses identified systems, institutional, community, and individual nurse and patient factors that helped or hindered care across 6 themes: profits over patients, care continuity and hospital-community partnerships, insufficient staffing and time constraints, technology to address language barriers, patients’ determinants of health, and individual nurses’ beliefs and backgrounds.

**Meaning:**

These findings suggest that nurses are informants of the strategies hospitals should use to advance high-quality, equitable care to socially disadvantaged patients.

## Teaser

This qualitative study examines hospital nurses’ perceptions of the various factors that help or hinder their ability to provide quality care to socially disadvantaged populations.

## Introduction

Disparities in outcomes for hospitalized patients living with substantial social disadvantages are long-standing and were exacerbated during the COVID-19 pandemic. During the pandemic, the risk of death was nearly 50% higher for members of socially disadvantaged communities, including those from minoritized racial and ethnic groups and people with limited economic resources.^1^ Mortality disparities for disadvantaged communities have been linked to systemic inequities,^2^ which are defined as the distribution of resources in ways that advantage some groups over others. For socially disadvantaged communities, this is reflected in systemic differences in hospitals where they receive care.^1,3^

Prior studies have established that hospitals caring for the most socially disadvantaged patients differ in many respects, and decades of research have demonstrated a link between the unequal distribution of access to high-quality health care and disparate outcomes.^4^ Hospital size, teaching status, and patient volume are examples of structural hospital characteristics that have been related to the quality of treatment.^5,6^ Nursing resources, such as staffing levels, are also important hospital features that are known to vary considerably across US hospitals.^7^ Although these nursing-related organizational factors are important for the outcomes of socially disadvantaged patients cared for in hospital settings,^8,9,10,11^ less is known about the barriers nurses face when providing care to socially disadvantaged patients.

Because socially disadvantaged patients have more constrained resources, including inadequate transportation, housing instability, or fewer material resources,^1^ their nursing needs are heightened during acute hospitalization, requiring more complex nursing care. If nurses are unable to meet the needs of socially disadvantaged patients, disparate outcomes may result owing to care deficits. In this study, we sought to identify what helps or hinders nurses from providing high-quality care to socially disadvantaged patients. We examine the open-text responses of nurses working in New York and Illinois hospitals and provide an overview of the barriers and facilitators expressed by frontline clinicians in addressing health-related social needs and summarize recommendations from direct care nurses.

## Methods

### Study Overview

This qualitative analysis is the second phase of a sequential explanatory (quantitative qualitative) mixed-methods^12^ study that sought to determine the characteristics of high-performing and low-performing hospitals during the COVID-19 pandemic. The quantitative phase used a benchmarking approach to identify hospitals with observed mortality rates that were significantly higher (ie, low-performing hospital) or lower (ie, high-performing hospital) than the mortality rates for a set of matched patients from other hospitals.^13^ We found that the average mortality rate in the high-performing hospitals was 16.2%, compared with 31.5% in the low-performing hospitals. Although there were few differences between high-performing and low-performing hospitals in static characteristics, such as teaching status, better nurse staffing levels and more supportive work environments were identified as central, modifiable features of high-performing hospitals. Socially disadvantaged patients were less likely to be hospitalized in high-performing hospitals with supportive nursing resources. The focus of the present study was to understand the perspectives of nurses working in the high-performing and low-performing hospitals to determine what helped or hindered their ability to provide care to socially disadvantaged patients.

### Data Source

Data were derived from the RN4CAST-NY/IL survey of registered nurses. The RN4CAST-NY/IL survey was a large email-based survey conducted between April and June 2021 that collected data on nurse demographics, nurse job outcomes, the quality of nurses’ work environments, and the quality and safety of patient care. Nurses self-reported race and ethnicity according to predefined categories in the survey. Race and ethnicity data were collected to assess the demographic diversity of the nursing workforce in New York and Illinois. Through a collaboration with the National Council of State Boards of Nursing, 100% of nurses licensed to work in New York and Illinois were invited to participate in the online survey. Among the 70 072 survey respondents, 13 421 identified as hospital-based nurses. The full methods for the RN4CAST-NY/IL are described elsewhere.^14^

In this study, our sample included nurses working in 25 hospitals previously identified as high performers and 33 identified as low performers on in-hospital mortality^13^ who responded to the open-text question, “What helps (or hinders) your ability to provide quality care to vulnerable populations? (e.g. low SES, housing insecurity/homeless, racial/ethnic minorities, immigrant, limited English proficiency)?” There was no character limit to the open-text responses, and the average character length among nurses was 112.

This study was approved by the institutional review board at the University of Pennsylvania. Nurses consented for participation upon beginning the RN4CAST-NY/IL survey. The Strengthening the Reporting of Observational Studies in Epidemiology (STROBE) and Consolidated Criteria for Reporting Qualitative Research (COREQ) reporting guidelines were followed. The Centers for Medicare & Medicaid Service’s Cell Suppression Policy^15^ prohibits data reporting if a category or cell size has fewer than 10 respondents. Therefore, values for American Indian, Alaska Native, or Native Hawaiian were listed as *not reported* in our study demographics. We acknowledge that these practices, as well as the reporting of Alaska Native and Native Hawaiian into a single race category, perpetuate the invisibility of indigenous populations.^16^

### Statistical Analysis

We used the Social Ecological Model (SEM) as the conceptual framework for our directed content analysis of open-text responses from nurses conducted between March and October 2024. The SEM^17,18,19,20^ is a public health framework and states that phenomena can be examined from multiple overlapping, interrelated levels that influence one another. The SEM provides a framework for analyzing the content of the data inductively and deductively, leading to the identification of study themes.^21^

Open-text responses were analyzed and stored in NVivo software version 10 (QSR International).^22^ Quotations were edited for typographical errors and clarity, and organizational names were deidentified. Four study authors (L.A., K.A., K.J.M, and M.B.C.) developed a codebook^23^ composed of deductive codes based on the following SEM levels: individual, institutional, community, and systems. Two authors (L.A. and K.A.) conducted an initial round of open coding^24^ using deductive codes. A second round of coding was completed with inductive codes added to the codebook. The study team double-coded 20% of the responses to resolve discrepancies and achieve an interrater reliability of greater than 0.70 with the codebook.^25^ One coauthor (L.A.) then coded the remaining responses.

Coders were blinded to whether responses came from the high-performing or low-performing hospitals. A summary of code frequency across the hospitals was generated for descriptive analysis (eTable 1 in Supplement 1). These codes were grouped into 15 categories at the individual, institutional, community, and systems levels of the SEM. Over several meetings, the study authors discussed the initial categories and collapsed them into 6 larger themes. After multiple line-by-line reviews of exemplary quotations and discussion of the themes, no new themes were identified, signaling data saturation.

Techniques to uphold rigor and trustworthiness^26,27^ involved the inclusion of a diverse team of study authors (by race and ethnicity, career stage, and insider or outsider status to nursing and health care) to conduct data analysis, triangulating exemplary quotations, member-checking with researchers outside of the direct team, and communicating reflexivity and/or positionality throughout the study. In alignment with mixed-methods, integration of our findings was accomplished using the Pillar Integration Process procedures outlined by Johnson and colleagues^28,29^ and resulted in a joint display (eTable 2 in Supplement 1). To analyze differences in nurse demographics across high and low performing hospitals, *t* tests were used for continuous variables and χ^2^ tests for categorical variables, with statistical significance set at 2-sided *P* < .05. R statistical software version 4.1.0 (R Project for Statistical Computing) was used for statistical analysis.

## Results

In this qualitative study and directed content analysis of open-text responses from 1084 nurses (mean [SD] age, 47.1 [18.2] years), most nurses were female (986 respondents [91.0%]), were staff or direct care nurses (765 respondents [70.6%]), and had at least a bachelor’s degree (968 respondents [89.6%]). They had a mean (SD) of 18.9 (14.0) years of experience as a nurse. With regard to race and ethnicity, 127 respondents were Asian (11.8%), 156 were Black or African American (14.5%), 89 (8.3%) were Hispanic, 693 were White (64.2%) and 97 (8.9%) were other races, which included write-in responses from nurses. Demographics self-reported by nurses are described in Table 1. Information on hospital characteristics is provided in eTable 3 in Supplement 1.

**Table 1.** Characteristics of Nurses in 58 High-Performing and Low-Performing Hospitals

Characteristic	Nurses, No. (%)			*P* value
	Total (N = 1084)	Low-performing hospital (n = 490)	High-performing hospital (n = 594)	
Age and experience				
Age, mean (SD), y	47.1 (18.2)	48.1 (12.9)	46.9 (21.6)	.30
Age missing	14 (1.3)	NR	NR	NA
Experience, mean (SD), y	18.9 (14.0)	19.3 (14.3)	18.7 (14.3)	.50
Gender				
Male	92 (8.5)	37 (7.6)	55 (9.3)	.50
Female	986 (91.0)	449 (91.6)	537 (90.4)	
Other	NR	NR	NR	
Missing	NR	NR	NR	
Race				
American Indian or Alaskan Native	NR	NR	NR	NA
Asian	127 (11.8)	64 (13.1)	63 (10.7)	.30
Black or African American	156 (14.5)	110 (22.5)	46 (7.8)	<.001
Native Hawaiian	NR	NR	NR	NA
White	693 (64.2)	257 (52.7)	436 (73.8)	<.001
Other race^a^	97 (8.9)	54 (11.1)	43 (7.3)	<.05
Missing	NR	NR	NR	>.99
Hispanic ethnicity	89 (8.3)	44 (9.0)	45 (7.6)	.50
Position				
Staff nurse or direct care nurse	765 (70.6)	336 (68.6)	429 (72.2)	.20
Nurse manager	75 (6.9)	42 (8.6)	33 (5.6)	.10
Advanced practice nurse	63 (5.8)	24 (4.9)	39 (6.6)	.30
Other position	181 (16.7)	88 (18.0)	93 (15.7)	.40
Education				
Hospital diploma or associate’s degree	113 (10.5)	49 (10.0)	64 (10.8)	.80
BSN only	652 (60.3)	292 (59.8)	360 (60.7)	.80
At least a BSN	968 (89.6)	439 (90.0)	529 (89.2)	.80
Missing	NR	NR	NR	.90

Abbreviations: BSN, bachelor’s of science degree in nursing; NA, not applicable; NR, not reported (owing to small cell size <10 as required by the Centers for Medicare & Medicaid Services Cell Suppression Policy).

^a^ Other race category included write-in responses from nurses (eg, multiple race and Filipino American). Column totals may not equal 100% due to missingness and/or rounding. The authorship team acknowledges that these limitations can exacerbate existing inequities in the reporting of indigenous populations.

Six themes described what nurses say help or hinder their ability to provide quality care to socially disadvantaged patients: (1) profits over patients, (2) care continuity and hospital-community partnerships, (3) insufficient staffing and time constraints, (4) technology to address language barriers, (5) patients’ determinants of health, and (6) individual nurses’ beliefs and backgrounds. Identified themes overlaid onto the SEM are described as follows and displayed with exemplary quotations in Table 2.

**Table 2.** Social Ecological Model, Themes, and Example Quotations

Social Ecological Model level and study theme	Example quotation
System	
Profits over patients	“The organization philosophy seems to promote earning and saving money over staff and patient safety and well-being. Doctors that are high earners can have multiple complaints yet staff and patients that are ignored with management stating, ‘What do you want us to do?’” (nurse from a low-performing hospital)
	“Our current healthcare system (for profit insurance that places obstacles in the path of patient care) is beyond shameful.” (nurse from a high-performing hospital)
	“The working settings now these days are more concerned about profit than caring. I get paid to perform a job, upper management care less to whose expense that job is completed.” (nurse from a low-performing hospital)
Community	
Care continuity and hospital-community partnerships	“We have a lot of community resources, and our social workers are fairly well connected to the community.” (nurse from a high-performing hospital)
	“Lack of available timely community resources. Cut through the red tape. We need closed loop processes in the communities to open up to follow up on our high-risk fearful vulnerable populations—social workers have been severely restricted in actionable options on behalf of their patients. The video language system has helped with language barriers in this time of limited visitor assistance. Some elderly patients are fearful of interacting with electronic equipment.” (nurse from a high-performing hospital)
	“Premature discharges for patients without insurance. Restriction of products to use when the patient has no insurance. Lack of responsible case managers to assist with appropriate discharge to the community. Lack of home care services available in the community.” (nurse from a low-performing hospital)
Institutional	
Insufficient staffing and time constraints	“Better staffing. They need so many resources and so much support that it is challenging. Take away time from other patients. However, you don’t want to neglect them because they need so much help, resources and support so they can stay out of the hospital.” (nurse from a high-performing hospital)
	“We do not have access to full time Spanish interpreters. We use phone interpretation and it’s subpar and dangerous.” (nurse from a high-performing hospital)
	“Time and lack of staff. We use electronic interpreters, and it takes forever to load. Maybe if I wasn’t juggling so much I could wait for it to boot up and use it.” (nurse from a low-performing hospital)
Technology to address language barriers	“Difficulty in using the Cyracom translation phones (long wait times for translators, broken/limited equipment). Limited staff to spend adequate time teaching patients with language barriers, have difficulty grasping the information, need much reinforcement, who are afraid and need emotional support.” (nurse from a low-performing hospital)
	“The translator phone and Lexicomp help me to provide quality time to vulnerable pop. I think we could all use more sensitivity training to vulnerable population especially since we are a predominantly white hospital.” (nurse from a high-performing hospital)
	“We do not have access to full time Spanish interpreters. We use phone interpretation and it’s subpar and dangerous.” (nurse from a high-performing hospital)
Individual	
Patient determinants of health	“I’m not sure—I guess the knowledge that they may not get the support they need at home once they’re discharged? It makes me feel like some things I teach them are gonna be useless, because they may not have the resources or support they need to carry out their own care at home. I try to treat all of my patients with the same level of care, with a little extra assistance to those who may not have the coping skills to deal with their personal situation.” (nurse from a high-performing hospital)
	“Lack of patient education from primary providers, patients are often under or misinformed when it comes to their care. This further hinders the care they need in the hospital setting. In labor and delivery many women come in with gestational hypertension and don’t understand the gravity of the situation, many times it’s due to a language barrier or a socioeconomic issue.” (nurse from a low-performing hospital)
	“The decreased trust that these populations have in healthcare, because they have either traditionally had their access limited to it or been treated poorly or dismissively by people in the healthcare professions.” (nurse from a high-performing hospital)
Individual nurses’ beliefs and backgrounds	“Sometimes the doctors don’t listen to minority patients on the pain level and their complaints as possible symptoms.” (nurse from a high-performing hospital)
	“This hospital has significant work to do in hiring/recruiting diverse staff. You can’t take great care of vulnerable populations when you don’t recognize the homogeneity of your own team.” (nurse from a high-performing hospital)

### SEM Level, System: Theme 1, Profits Over Patients

A systemic challenge that nurses described was the misalignment of financial incentives with the delivery of high-quality, equitable care to socially disadvantaged patients. Nurses in low-performing hospitals were more critical of hospital administration than those in high-performing hospitals regarding the prioritization of profits over patient-centered care: “The working settings now these days are more concerned about profit than caring. I get paid to perform a job, upper management cares less to whose expense that job is completed” (nurse from a low-performing hospital). Nurses in both types of hospitals described a systemic bias against minoritized populations that degraded clinician well-being: “Our hospital has implicit bias in respect to minorities, indigent, and patients with mental illness. Also, my hospital has a deference to ‘VIPs’ and donors, and it completely evaporates staff morale when we are told a VIP is coming in and we need to attend to them first or more conscientiously” (nurse from a high-performing hospital).

### SEM Level, Community: Theme 2, Care Continuity and Hospital-Community Partnerships

Across both types of hospitals, nurses emphasized the crucial role of social workers connecting patients with community resources. Nurses in high-performing hospitals more frequently expressed concerns surrounding patient care continuity. One nurse stated, “A lot of our patients are homeless and/or unfunded. They get great care, and our social workers are excellent at finding them placement” (nurse from a high-performing hospital). Nurses in both types of hospitals discussed positive outcomes from social service departments, with nurses in low-performing hospital providing more-specific examples of programs that helped socially disadvantaged populations: “Case management at our organization does a wonderful job with outpatient services/resources to help patients get the care they need. We also have a program in certain ‘high risk’ zip codes where Paramedics in the community follow up with patients after discharge and makes sure patients are getting what they need” (nurse from a low-performing hospital).

### SEM Level, Institutional

#### Theme 3: Technology to Address Language Barriers

Nurses in both types of hospitals stated that the presence and quality of language access technology helped provide high-quality care to patients with limited English proficiency: “The video language system has helped with language barriers in this time of limited visitor assistance. Some elderly patients are fearful of interacting with electronic equipment” (nurse from a high-performing hospital). Nurses in low-performing hospitals differed in that they more frequently discussed an issue of a language barrier or mentioned limited English proficiency as a hindrance: “Their limited English, their lack of understanding/being educated on what’s going on with them” (nurse from a low-performing hospital). In contrast, nurses in high-performing hospital mentioned issues driven by access to interpreter services and equipment comparatively more often “Difficulty finding interpreters for less common languages (i.e. Taishanese, Urdu, Fouzhu)” (nurse from a high-performing hospital).

#### Theme 4: Insufficient Staffing and Time Constraints

In both high-performing and low-performing hospitals, nurses described how high workloads and time constraints hindered their ability to provide quality care to socially disadvantaged populations. Staffing was one of the most common themes discussed in the data. Nurses in high-performing hospitals specifically discussed issues of time constraints more: “It takes TIME to educate. But then there is the pressure of tending to other patients’ actions—again that would be staffing. So, it is difficult to improve medical care in the long run” (nurse from a high-performing hospital). In contrast, nurses in low-performing hospitals more often mentioned concerns with staffing levels: “Stretched so thin due to inadequate staffing that we do not have enough time to determine what patient’s needs are and care for them based on their needs other than medical” (nurse from a low-performing hospital).

### SEM Level, Individual 

#### Theme 5: Patient Determinants of Health

Nurses described patients’ health literacy, family support, and demographic characteristics as factors impacting the care of socially disadvantaged populations. Nurses in high-performing hospitals more often mentioned how prior interactions with health care resulted in a lack of trust among patients from socially disadvantaged backgrounds: “Once patients leave the hospital there are all sorts of ways they fall through the cracks. I have had experience as an active RN of patients discharged home on new insulin and the patient has no clue how to use it. Or patients sent home on g-tube feedings and the people caring for them also, have no clue” (nurse from a high-performing hospital). “We use a translation system on an iPad, but sometimes it’s the level of literacy that makes it hard to ensure patients have a full understanding of their health and follow up care” (nurse from a low-performing hospital).

#### Theme 6: Individual Nurses’ Beliefs and Backgrounds

Nurses described how their own personal life experiences influenced their beliefs and the care of patients from socially disadvantaged backgrounds. However, nurse responses from both types of hospitals reflected bias, both from themselves and other staff, including nurse and physician colleagues: “Low socioeconomic status and racism from our providers” (nurse from a low-performing hospital). “Limited resources for undocumented patients—not sure why we allow these patients to continue to live here with no consequence and we have to continue to use our tax dollars to take care of them. Frustrated when you see elderly patients not being able to afford medication but then we give free health care to people who haven’t paid a dime in taxes” (nurse from a high-performing hospital).

Nurses in both types of hospitals described treating patients equally, and the content surrounding treating patients equally was more often discussed among nurses from high-performing hospitals: “I work at a very large NYC hospital and I can honestly say your care does not differ if you are black, white, homeless or living in a penthouse as a billionaire. All patients truly get the same nursing care” (nurses from a high-performing hospital). “Nothing, all my patients are treated equally based on their needs and that’s my philosophy, socioeconomic status is not a factor” (nurse from a low-performing hospital).

Nurses in high-performing hospitals more commonly expressed a need for increased training in cultural competency, using terms such as *diverse/cultural education*, *sensitivity training*, and *cultural sensitivity*. Nurses in both types of hospitals emphasized the need for a more diverse workforce that reflects the patient populations served: “I believe some nurses still need to be trained on how to be more sensitive and not to prejudge people; for example that are homeless or are obviously struggling with drug addiction. I see how these vulnerable populations are treated differently which should not be the case and wish that this can be improved by sensitivity training” (nurse from a high-performing hospital) (Table 3).

**Table 3.** Nurse-Informed Strategies to Advance Equitable Care to Socially Disadvantaged Patients

Social Ecological Model level and theme	Strategy	Nurse perspective
System		
Profits over patients	Invest in funding for safety net and social programs Reduce administrative and resource barriers to care continuity Offer competitive offers to hire a strong, committed health care workforce Outward advocacy and action to address health disparities on behalf of hospital leaders	“Things that help: government programs that are designed to reach out to these vulnerable patients with both funding and administrative support to implement the proposed initiatives; jobs that are competitive in terms of wages/benefits in order to attract stronger candidates who want to help these vulnerable populations.” (nurse from a low-performing hospital) “Cut through the red tape. We need closed loop processes in the communities to open up to follow up on our high-risk, fearful vulnerable populations. Social workers have been severely restricted in actionable options on behalf of their patients.” (nurse from a high-performing hospital) “Acknowledgement that health disparities exist, and we should allocate resources to help those in need. Sometimes it can be our own coworker that needs some extra help but is too afraid to say anything. There should be a way for employees to anonymously ask for help or support if needed.” (nurse from a high-performing hospital)
Community		
Care continuity and hospital-community partnerships	Offer community-based programs and partnerships that bridge the acute care and outpatient or discharge experience Increase social work availability and resources	“Our hospital has been very innovative in trying to meet those need (i.e., food insecurity—we can provide 2 types of food packages for the patient to bring home and have partnered with community resources; domestic violence/human trafficking—we have a dedicated group to assist those in this situation; SANE nurses for rape cases; housing for homeless who can demonstrate a desire to get back on their feet incorporating job training, financial counseling, counseling, legal services).” (nurse from a low-performing hospital) “Readily available social work and case management staff improve responsiveness to particular population challenges. Close collaboration between social work and medical teams to streamline and enhance discharge planning. Nurse coaching to patients improves patient-to-doctor communication and helps patients center convey their needs.” (nurses from a high-performing hospital)
Institutional		
Insufficient staffing and time constraints	Invest in safe patient-to-nurse staffing ratios Staff a sufficient number of social workers on health care units	“Staffing. More RNs are desperately needed.” (nurse from a high-performing hospital) “We need a better staff to patient ratio.” (nurse from a low-performing hospital) “Also we need more nurses! Period!” (nurse from a high-performing hospital) “City-wide services are not available for the homeless and our regular alcoholics these can be problems that have no direct answer but we need more social workers to help this vulnerable population.” (nurse from a low-performing hospital)
Technology to address language barriers	Adequately resource hospitals with language access services (interpreters) Provide multiple modalities of language services to accommodate the range of patient populations (eg, hearing impaired) such as video services, in-person interpreters, and translated documents	“Ability to have instantaneous access to interpreters/translators and patient education materials in multiple languages.” (nurse from a high-performing hospital) “In person interpreters because confused and old patients can’t use the video or telephone interpreters.” (nurse from a low-performing hospital) “Need for more discharge instructions and patient literature to be made available in written Spanish or other languages.” (nurse from a high-performing hospital)
Individual		
Patient determinants of health	Expand outpatient mental health resources and services Provide trainings and resources for families to support loved ones at home Invest in transitional care programs and resources that address the social determinants of health Expand community engagement and patient outreach	“Long term mental health facilities are being closed when I feel we need more in order to provide patients struggling from mental illness with the help they need to live a more purposeful and fulfilling life.” (nurse from a high-performing hospital) “Many families need therapy and training to deal with patients when they return home.” (nurse from a high-performing hospital) “We gave a large population of homeless patients who need help and have nowhere to go and the upper managers don’t want them waiting for shelters in the waiting area due to other patient satisfaction! There needs to be a place for them to wait or sleep without disturbing the other patients!” (nurse from a low-performing hospital) “We need more mental health workers and facilities to deal with the various mental health and drug addiction problems. We need access to vaccinations for our vulnerable populations and for them to actually take the vaccines. We need to, as a community, deal with the fact we have a racial problem in our community and to improve access to health food, healthcare and have racial justice in our community. We have gangs and gun violence in our community, find ways to deal with those issues.” (nurse from a low-performing hospital) “The lack of trust in the medical system, social media spreading false conspiracy theories, lack of education, financial security and quality health care insurance, cultural beliefs and practices, I feel are elements that can potentially hinder RNs ability to provide quality care.” (nurse from a high-performing hospital)
Individual nurses’ beliefs and backgrounds	Diversify the clinician workforce Provide education to clinicians on experiences of minoritized populations such as the LGBTQ community, people who use drugs, and the unhoused Develop and disseminate cultural competency and sensitivity trainings	“What helps with ethnic minorities and immigrants is having a multilingual workforce.” (nurse from a low-performing hospital) “Limited staff diversity and lack of bilingual staff. Diversity/cultural education to improve understanding of socioeconomic and racial and cultural needs of the community we serve.” (nurse from a high-performing hospital) “I’d say what helps is that I’m mostly bilingual, I’m Hispanic, and that helps me provide quality care to Spanish speaking patients if I encounter them. Recently I’ve discovered that I belong in the LGBTQ community as well. What would hinder my ability to provide care to the LGBTQ community would be not being able to be my authentic self at work. Visibility matters. Not enough educational opportunities on LGBTQ and transgender health, mental health, immigrant health would also hinder myself and coworkers’ ability to provide quality care.” (nurse from a high-performing hospital) “Language services help as well as training on more vulnerable populations. In person training would be more effective in my opinion, versus the electronic training done on the computer.” (nurse from a low-performing hospital) “I believe some nurses still need to be trained on how to be more sensitive and not to prejudge people for example that are homeless or are obviously struggling with drug addiction. I see how these vulnerable populations are treated differently which should not be the case and wish that this can be improved by sensitivity training.” (nurse from a high-performing hospital)

Abbreviations: LGBTQ, lesbian, gay, bisexual, transgender, and queer; RN, registered nurse.

## Discussion

In this qualitative study using directed content analysis of survey responses from nurses in high-performing and low-performing hospitals, nurses described what helped or hindered care to socially disadvantaged patients, including hospital financial priorities, hospital-community partnerships, nurse staffing ratios, language access services, patient determinants of health, and nurses’ beliefs and backgrounds. Our findings are novel in that we identify strategies to advance equitable care in hospitals through responses from nurses, the most abundant health care professional group.

There was overwhelming agreement among nurses in both high-performing and low-performing hospitals that investments in safer nurse staffing ratios and a diverse, multilingual clinician workforce are needed to support socially disadvantaged patients. In addition, nurses in both groups identified substantial barriers to the delivery of equitable care to patients with limited English proficiency and populations in need of posthospitalization care, such as those experiencing serious mental illness, substance use disorder, and housing instability. There were distinctions between nurses in high-performing and low-performing hospitals related to the frequency of discussing profit-based health care practice, community-based programming, and the need for strong language access services.

Nurses across hospitals described how limited resources in their work environment contribute to suboptimal outcomes for patients with substantial social needs, which is supported by over 15 years of evidence.^8,11,30,31^ Studies demonstrate that hospitals with better nursing resources, such as high-quality nurse work environments characterized by adequate staffing resources, nurse-physician collegiality, nurse autonomy, nurse integration in hospital decisions, and foundations for quality care,^32,33^ are associated with diminished disparities for patients from socially disadvantaged neighborhoods.^1^ Collectively, results from these studies demonstrate how nursing-related organizational factors are associated with inequitable patient outcomes. The current study extends these quantitative findings through the addition of rich qualitative responses and, thus, expands the scope and breadth of our results. Some areas such as staffing and work environments are confirmed through the open-text responses of nurses as key areas of focus. Other areas that were not included in prior surveys of nurses, such as the need for further cultural sensitivity training, more care coordination and continuity, the importance of language access services, and greater workforce diversity were identified through only our qualitative analysis.

Collectively, nurses in our study provided recommendations for improving care delivery and outcomes for socially disadvantaged patients (Table 3). Nurses recommended reliable and high-quality language access services and equipment to provide equitable communication to individuals with language needs, such as those with limited English proficiency, elderly individuals, and individuals with auditory and visual impairment. Nurses also outlined the need for hospitals to develop, expand, and promote programs that address the social determinants of health, such as legal services, housing support, food packages, and resources for those experiencing interpersonal violence.

Staffing was one of the most frequently discussed topics by nurses in our study, underscoring a key area of intervention to improve care for socially disadvantaged patients. Hospitals in the US have made progress in enacting policies to increase the number of nurses at the bedside through safe staffing mandates. California and Oregon are 2 states that have passed legislation mandating safe patient-to-nurse staffing ratios^34^ in hospitals (ie, 4 patients per every 1 nurse in emergency departments). More states have introduced similar legislation given substantial evidence demonstrating that patients benefit from staffing ratios by receiving more direct care nursing hours.^35,36,37^

Nurses recommended cultural sensitivity training for health care professionals about implicit bias and experiences of historically marginalized groups such as the LGBTQAI (lesbian, gay, bisexual, transgender, queer, asexual, and intersex) community. Organizational interventions such as Magnet Recognition^38^ provide structures for hospitals to cultivate a supportive culture of nursing care through clinical practice, research, and leadership. As of 2023, Magnet hospitals are required to demonstrate evidence of education and training in diversity, equity, inclusion, and cultural competence. Although most hospitals require sensitivity training, such modalities are often onetime, virtual offerings. The responses of nurses in our study demonstrate that on-going continuing education about a wider range of patient populations is desired.

### Limitations

This study has limitations that should be mentioned. Our study analyzed data that were collected from nurses working in hospitals in 2 large states during the COVID-19 pandemic, which may limit generalizability. However, studies conducted before the pandemic found that nurses were already experiencing substantial resource constraints, such as inadequate staffing, that negatively affected their ability to care for socially disadvantaged patients.^7,39^ Given that the write-in responses were optional in the survey, our data may have a response bias preferencing participants with a particular interest in the topic of care for the socially disadvantaged. A strength of our data, however, is that we received over 1000 responses to the question, including balanced perspectives of nurses on facilitators, hindrances, and biases related to the care of socially disadvantaged patients.^40^

## Conclusions

In this qualitative study of 1084 open-text responses, nurses in high-performing and low-performing hospitals described what helped or hindered care for socially disadvantaged patients across 6 study themes. Facilitators to care included high-quality language access services, hospital-community partnerships, and a diverse, multilingual health care workforce. Barriers included insufficient nurse staffing, a profit-focused health care system, and cultural biases toward patients by clinicians. Hospitals should consider using the strategies identified by the nurses to improve care for socially disadvantaged patients.

## Notes

Supplement 1. **eTable 1.** Summary of Code Frequencies Among Nurses in High and Low Performing Hospitals **eTable 2.** Mixed-Methods Integration **eTable 3.** Hospital Characteristics

Supplement 2.
Data Sharing Statement
