# Supplementary material for: Hospital Nurse Perspectives on Barriers and Facilitators to Caring for Socially Disadvantaged Patients
Source: JAMA Netw Open. 2025 Jun 6;8(6):e2512397. doi: 10.1001/jamanetworkopen.2025.12397 (PMC12144619; doi:10.1001/jamanetworkopen.2025.12397)
Supplement: Supplement 1. — eTable 1. Summary of Code Frequencies Among Nurses in High and Low Performing Hospitals eTable 2. Mixed-Methods Integration eTable 3. Hospital Characteristics [file jamanetwopen-e2512397-s001.pdf]

Supplemental Online Content

Brooks Carthon JM, Muir KJ, Ang L, et al. Hospital nurse perspectives on barriers and facilitators to caring for socially disadvantaged patients. *JAMA Netw Open*. 2025;8(6):e2512397. doi:10.1001/jamanetworkopen.2025.12397

eTable 1. Summary of Code Frequencies Among Nurses in High and Low Performing Hospitals

eTable 2. Mixed-Methods Integration

eTable 3. Hospital Characteristics

This supplemental material has been provided by the authors to give readers additional information about their work.

© 2025 Brooks Carthon JM et al. *JAMA Network Open*.

eTable 1: Summary of Code Frequencies Among Nurses in High and Low Performing Hospitals

	High-performing hospital nurse responses (N)	HP % (row)	Low performing hospital nurse responses (N)	LP % (row)	Total (1084)
**Individual level**	223	52%	205	48%	428
Patient education, views, values	70	57%	53	43%	123
Patient demographics, characteristics	69	44%	88	56%	157
Nurse characteristics, views	57	56%	45	44%	102
Equal treatment	27	59%	19	41%	46
**Institutional level**	466	54%	391	46%	857
Equipment and technology	75	60%	51	40%	126
Language and interpretation services	149	58%	110	42%	259
Staffing, workplace collaboration, environment	98	47%	111	53%	209
Time constraints	48	68%	23	32%	71
Organizational type	27	47%	30	53%	57
Language Barrier	69	51%	66	49%	135
**Community Level**	144	57%	108	43%	252
Continuity of care	68	61%	43	39%	111
Social services, community support and resources	76	54%	65	46%	141
**Systemic, All Levels**	57	47%	65	53%	122
Systemic healthcare issues	31	41%	44	59%	75
Insurance	26	55%	21	45%	47
**Other**					
Cultural competency and training	19	59%	13	41%	32
total	**909**	**54%**	**782**	**46%**	**1691**

Note. N indicates number of nurse responses. HP=high performing hospital. LP=low performing hospital. Table includes levels from the Social Ecological Model (e.g., "Individual Level") and 15 categories emerging from the inductive and deductive coding of the data that informed the data analysis (see "Methods" in-text).

© 2025 Brooks Carthon JM et al. *JAMA Network Open*.

eTable 2: Mixed-Methods Integration

**Table 3. Pillar Integration of Mixed Methods Results**				
**QUANT Data**	**QUANT Categories**	**PILLAR BUILDING THEMES**	**QUAL Categories**	**QUAL Data**
Mean patient-to-nurse ratios in medical-surgical units (HPs=5.4 vs. LPs=6.1, p<0.001).	Lower-performing hospitals cared for significantly higher ratios of high-to-low SVI patients.	Nursing resources are a unifying factor that facilitate advancing high quality care to socially disadvantaged patients	Unsafe patient to nurse staffing ratios deterred nurses from spending sufficient time identifying and addressing the needs of socially disadvantaged patients<br><br>Nurses' concerns about unsafe workloads are not addressed by hospital management	**Insufficient Staffing and Time Constraints Barriers**
Mean Practice Environment Scale-Nursing Work Index score (HPs=2.78 vs. LPs=2.58, p<0.001)	Nursing factors were a key differentiating factor of high and low performing hospitals. High-performing hospitals had higher quality work environments (i.e., administration that listens and responds to employee concerns; a nurse manager who is a good manager and leader; a lot of teamwork between nurses and physicians; enough staff to get the work done; and a clear philosophy of nursing that pervades the patient care environment)			
Ratio of high-to-low SVI patients (HPs=1.0 vs. LPs=6.1, p<0.001)	Lower-performing hospitals cared for significantly higher ratios of high-to-low SVI patients.	Nurse staffing measures must capture nuanced characteristics of patient demographics such as the SDOH and social vulnerability in addition to clinical acuity to ensure workforce	Patients' health literacy, family support, and demographic characteristics as factors impacting the care of socially disadvantaged populations.	**Patient Determinants of Health**

© 2025 Brooks Carthon JM et al. *JAMA Network Open*.

		capacity and quality care		
**QUANT Data**	**QUANT Categories**	**PILLAR BUILDING THEMES**	**QUAL Categories**	**QUAL Data**
High-technology procedure capabilities (HPs=72.0% vs. LPs=39.4%, p=0.03)	High-performing hospitals were more likely to have high technology capabilities, such as performing organ transplants and open-heart surgery.	Measures of technology capabilities should be expanded to include language access services, equipment, and availability	Insufficient or unreliable language access services (e.g., interpreters, video language lines)	**Technology to Address Language**
Teaching hospital (HPs=68.0% vs. HPs=84.8%, p=0.37)<br><br>Number of beds (HPs=574.4 vs. LPs=388.8, p=0.09)<br><br>Proportion of baccalaureate-prepared nurses (HPs=0.84 vs. LPs=0.79, p=0.06)<br><br>Number of full-time physicians (HPs=265.3 vs. LPs=147.1, p=0.22)	Other hospital structural characteristics, such as teaching status, number of beds, proportion of bachelor's prepared nurses, and number of full-time physicians were not statistically different between high and low performing hospitals.	The structural characteristics of hospitals must be expanded to include community engagement, social responsibility, and the number of programs addressing patient SDOHs.	The extent to which hospitals provide community-engaged programs that provide care continuity services	**Care Continuity and Hospital-Community Partnerships**
Not identified in quantitative findings	N/A	Need for diverse and multilingual healthcare workforce and high quality trainings in the SDOH, implicit bias, and experiences of minoritized populations	Nurses observe and report racial and cultural biases towards socially disadvantaged patients. The diverse backgrounds of nurses are a strength in	**Individual Nurses' Beliefs and Backgrounds**

© 2025 Brooks Carthon JM et al. *JAMA Network Open*.

			providing equitable care.	
Not identified in quantitative findings	N/A	Health system practices, policies, actions, mission and values, must align with the needs of communities, patients, and frontline clinicians	Nurses describe preferential treatment of specific populations based on socioeconomic status and financially driven values	**Profits over Patients**

Notes: HP=High-performing hospital; LP=low-performing hospital. Quant data and categories were published previously.[12] The combined quantitative and qualitative result are displayed in five columns using the Pillar Integration Process. Four steps make up this methodical approach: listing, matching, checking, and pillar building. The results of the previous quantitative phase of the study were listed first (QUANT data column). This included the percentage of the overall mortality in high and low performing hospitals. The QUANT categories column then provided a thematic summary of the overall quantitative data. The principal topics (QUAL categories column) and qualitative subthemes (QUAL data column) were then enumerated and arranged horizontally according to comparable QUANT content. Next, the qualitative subthemes (QUAL data column) and major themes (QUAL categories column) were listed and organized in a manner that matched with similar QUANT content horizontally in the joint display. Finally, the pillar building stage (Pillar Building Themes column) allowed for the identification of overarching themes, patterns, and shared areas of agreement (and disagreement) across data types.

© 2025 Brooks Carthon JM et al. *JAMA Network Open*.

eTable 3: Hospital Characteristics

	All study hospitals (n=96)		Low-performing hospitals (n=33)		High-performing hospitals (n=25)		P-value
Hospital Characteristics	***n***	***%***	***n***	***%***	***n***	***%***	
**Teaching status**							0.4
None/missing	31	32.3	5	15.2	8	32.0	
Minor	24	25.0	11	33.3	6	24.0	
Major	41	42.7	17	51.5	11	44.0	
**High technology capability**							<.05
No/Missing	44	41.8	20	60.6	7	28.0	
Yes	52	54.2	13	39.4	18	72.0	
**State**							0.4
Illinois	41	42.7	11	33.3	11	44.0	
New York	55	57.3	22	66.7	14	56.0	
**Ownership**							
Not-for-profit: Government nonfederal	13	13.5	8	24.2	1	4.0	0.1
Not-for-profit: non-government	76	79.2	22	66.6	22	88.0	
For-profit	2	2.0	1	3.0	0	0.0	
Missing	5	5.2	2	6.0	2	8.0	
	M	***SD***	***M***	***SD***	***M***	***SD***	
**Number of beds**	443.3	336.3	388.8	168.4	574.4	500.2	0.1
**Number of COVID-19 patients**	767.7	670.5	590.9	303.3	1046.2	965.9	<.05

Note. M=Mean; SD=Standard deviation. Missing data were noted for teaching status (n=5 hospitals in full sample; 2 low-performing and 2 high-performing) and high technology capability (n=15 hospitals in full sample; 8 low-performing and 3 high-performing). Percentages may not total 100% due to missingness and rounding.

© 2025 Brooks Carthon JM et al. *JAMA Network Open*.
