# Supplementary material for: Hospital Nurse Perspectives on Barriers and Facilitators to Caring for Socially Disadvantaged Patients
Source: JAMA Netw Open. 2025 Jun 6;8(6):e2512397. doi: 10.1001/jamanetworkopen.2025.12397 (PMC12144619; doi:10.1001/jamanetworkopen.2025.12397)
Supplement: Supplement 2. — Data Sharing Statement [file jamanetwopen-e2512397-s002.pdf]

Data Sharing Statement

Brooks Carthon. Hospital Nurse Perspectives on Barriers and Facilitators to Caring for Socially Disadvantaged Patients. *JAMA Netw Open*. Published June 06, 2025. doi:10.1001/jamanetworkopen.2025.12397

Data
Data available: No
